# 3D Printing as a Strategy to Scale-Up Biohybrid Hydrogels for T Cell Manufacture

**DOI:** 10.1021/acsami.4c06183

**Published:** 2024-09-17

**Authors:** Eduardo Pérez Del Río, Sergi Rey-Vinolas, Fabião Santos, Miquel Castellote-Borrell, Francesca Merlina, Jaume Veciana, Imma Ratera, Miguel A. Mateos-Timoneda, Elisabeth Engel, Judith Guasch

**Affiliations:** † Department of Molecular Nanoscience and Organic Materials, Institut de Ciència de Materials de Barcelona (CSIC), Campus UAB, Bellaterra 08193, Spain; ‡ Centro de Investigación Biomédica en Red de Bioingeniería, Biomateriales y Nanomedicina (CIBER-BBN), Madrid 28029, Spain; § IMEM-BRT Group, Department of Materials Science and Engineering, EEBE, Technical University of Catalonia (UPC), Barcelona 08019, Spain; ∥ Institute for Bioengineering of Catalonia (IBEC), The Barcelona Institute of Science and Technology (BIST), Barcelona 08028, Spain; ⊥ Dynamic Biomaterials for Cancer Immunotherapy, Max Planck Partner Group, ICMAB-CSIC, Campus UAB, Bellaterra 08193, Spain; # Bioengineering Institute of Technology, Universitat Internacional de Catalunya (UIC), Sant Cugat del Vallès 08195, Spain

**Keywords:** 3D printing, 3D hydrogels, T cells, cell therapy, cancer

## Abstract

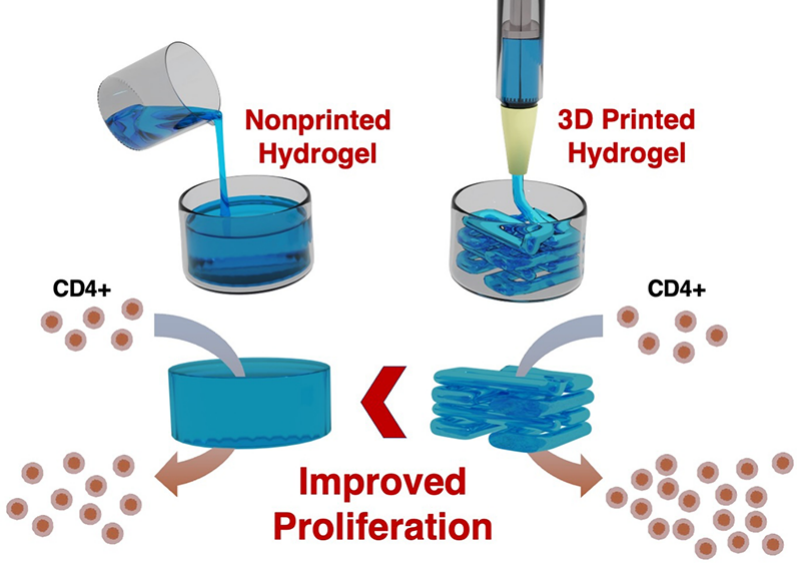

The emergence of cellular immunotherapy treatments is introducing more efficient strategies to combat cancer as well as autoimmune and infectious diseases. However, the cellular manufacturing procedures associated with these therapies remain costly and time-consuming, thus limiting their applicability. Recently, lymph-node-inspired PEG–heparin hydrogels have been demonstrated to improve primary human T cell culture at the laboratory scale. To go one step further in their clinical applicability, we assessed their scalability, which was successfully achieved by 3D printing. Thus, we were able to improve primary human T cell infiltration in the biohybrid PEG–heparin hydrogels, as well as increase nutrient, waste, and gas transport, resulting in higher primary human T cell proliferation rates while maintaining the phenotype. Thus, we moved one step further toward meeting the requirements needed to improve the manufacture of the cellular products used in cellular immunotherapies.

## 1 Introduction

Adoptive cell (immuno)therapy (ACT) consists of using (autologous) T cells to mediate tumor or pathogen destruction or even fight against immune diseases.^1−5^ In oncology, T cells are directly selected from the tumor or genetically modified to recognize it, cultured, and expanded in vitro, and finally reintroduced to the patient. In addition to the radical change that supposes using living cells as therapeutic agents in comparison with current drugs, they have the capacity to adapt their response to the stimuli encountered as well as to provide long-term protection.^6−9^ Nevertheless, the manufacturing of clinical doses of persistent therapeutic T cells can be technically challenging and economically expensive,^10^ thus limiting the translation of ACT to the clinics.^11−13^

Nowadays, the method used to obtain the immune cells needed for these therapies usually consists of culturing the cells, normally T cells, with artificial antigen-presenting cells (APCs), such as MACS MicroBeads (Miltenyi, Germany) or Dynabeads (Thermo Fisher Scientific, USA), to mimic the immunological synapse,^14^ in suspension using bioreactors. However, efforts are being devoted to reproducing the extracellular matrix (ECM) of secondary lymphoid organ tissue, especially that of the lymph nodes (LNs), as this is where the activation of T cells by APCs naturally occurs.^15^ Indeed, there is growing evidence about the influence of this immune microenvironment on the resulting cell products.^16−21^

Both natural and synthetic hydrogels have been used to recreate the ECM of different tissues with different objectives, such as broadening the current knowledge in cell biology or culturing cells in an environment that resembles the human body better than the conventional culture recipients.^22^ 3D PEG–heparin hydrogels have previously been shown to improve primary human T cell proliferation and influence the resulting cell phenotypes.^17,18^

3D printing permits the automated fabrication of 3D objects in a layer by layer approach of complex structures with precisely designed geometries.^23^ Among the different types of 3D printing technologies, extrusion-based printing is especially popular in biomedicine, as it simply consists of extruding a material through a nozzle and depositing it in filaments on a platform to form a 3D structure.^24^

**Figure 1:**
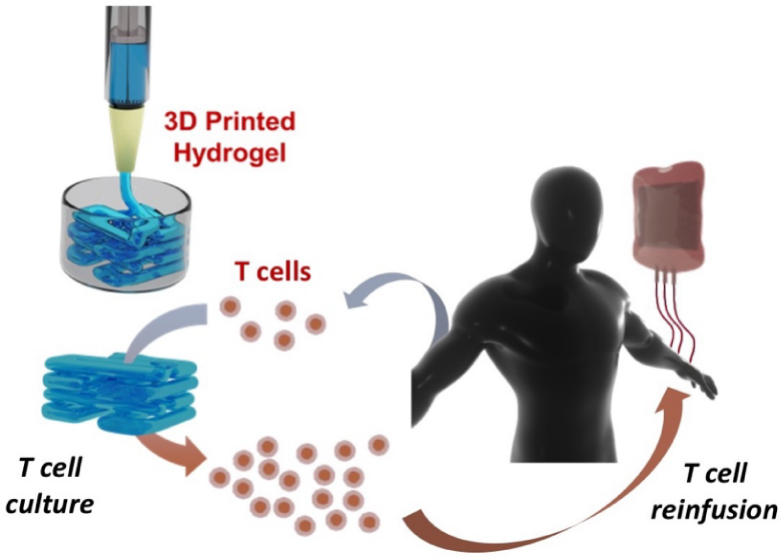
Simplified scheme of autologous T cell product fabrication using a 3D printed hydrogel for cell culture.

With the objective of moving one step forward in fabricating artificial LNs and helping to overcome the current limitations of ACT related to the manufacturing of large amounts of therapeutic T cells, the previously described PEG–heparin hydrogels were analyzed as ink for 3D printing and used for human T cell culture (Figure 1).

## 2 Materials and Methods

### 2.1 Materials

4-arm thiol terminated poly(ethylene oxide) with *M*_w_ = 10000 Da was purchased from Nanosoft Biotechnology LLC (USA). Unfractionated heparin with average *M*_w_ = 15000 Da was acquired from Thermo Scientific Chemicals (USA) and functionalized with maleimide as previously described,^17^ which is a protocol adapted from prior publications.^25,26^

Thermo Fisher Scientific (USA) provided penicillin/streptomycin (P/S), Dynabeads, fetal bovine serum (FBS), and the CellTrace CFSE cell proliferation kit. Miltenyi Biotec GmbH (Germany) provided the CD4+ T cell isolation kit, while Stemcell Technologies (Canada) provided Lymphoprep. Regarding flow cytometry, antihuman CD62L PE and its control were acquired from BioLegend (USA), while antihuman CD4 PE, CD3 FITC, CD45RO FITC, and their controls were bought from Immunotools GmbH (Germany). For 3D printing, the syringes used were from Nordson EFD (USA). RPMI-1640 media, Dulbecco’s phosphate-buffered saline (PBS), and any other nonmentioned products were purchased from Merck (USA).

### 2.2 3D Printing of PEG–Heparin Hydrogels

3D printing experiments were performed using a 3D Discovery printer (RegenHU Biosystem Architects, Switzerland). One day before printing, sterilized solutions of 4-arm thiolated PEG and maleimide-functionalized heparin were mixed in a sterile syringe suitable for printing. The mixture was maintained overnight at room temperature. Afterward, a TIP27GA TT 008’’ NAT tip was used to place the syringe in the printer, and printing was achieved at 1.2 bar of pressure and 15 mm/s of printing speed. For the scaffolds with a higher material/cell ratio, the tip used was the TIP25GA TT 010’’ and operated at a pressure of 1.6 bar and a printing speed of 10 mm/s. Nine segments were 3D printed with each needle to determine the filament diameter and spreading ratio using ImageJ. The filament diameter was measured at 10 different random positions, and the spreading ratio was obtained by dividing the measured diameter by the internal diameter of the needle.^27,28^ The 4/6 layer grids consisted of lines spaced 1.5 mm apart and were 4/6 layers in height, organized in a squared shape. To obtain larger grids of 10 layers in height, a modification of the structure was necessary to increase its stability, which was achieved by designing a circular grid with each layer arranged perpendicularly in an alternating manner (a separation of 1.5 mm between lines was maintained).

Bulk (non-printed) PEG–heparin hydrogels were produced at a concentration of 3% of PEG in weight and a ratio of PEG/heparin of 1:1.5, by mixing a PBS solution of 4-arm thiolated PEG with a solution of maleimide-functionalized heparin in the same buffer.

### 2.3 Primary T Cell Culture Using PEG–Heparin Hydrogels

Primary human CD4+ T cells were isolated from buffy coats of adult donors collected by “Banc de Sang i Teixits” (Barcelona, Spain), after ethical approval was obtained from the Research Ethics Committee of the Autonomous University of Barcelona (Nr. 5099). The CD4+ T cells were isolated from peripheral blood mononuclear cells using density gradient centrifugation with Ficoll, combined with a commercial CD4+ T cell isolation kit, following an established protocol.^17−19,29,30^ Flow cytometry was used to check cell purity using antihuman CD3 FITC and CD4 PE (with their corresponding negative controls). Only CD3+CD4+ T cells > 90% (usually >95%) were considered for experiments. After the purification, cells were maintained in RPMI medium (10% FBS + 1% P/S) in an incubator at 37 °C until their seeding. At this point, they were added on top of PEG–heparin hydrogels, as their pore size and interconnectivity allow adequate cell infiltration. Cells are seeded at a concentration of 1 × 10^6^ cells/ml together with Dynabeads in a 1:1 ratio.

### 2.4 Primary T Cell Differentiation and Proliferation in PEG–Heparin Hydrogels

Primary human CD4+ T cells were stained with a CFSE cell proliferation kit before seeding according to the instructions of the manufacturer for proliferation studies. On day 6, they were examined by flow cytometry after thorough pipetting to destroy the hydrogels and maximize cell recovery. To diminish the intrinsic donor variability, the proliferation results were normalized to the positive control of each donor. To obtain the cellular phenotypes, primary human CD4+ T cells were measured 5 days after seeding, following the methodology described above to recover the cells. They were stained with antihuman CD45 RO FITC and CD62L PE (and the corresponding negative controls) for 30 min at 0 °C. Finally, the cells were washed and examined by flow cytometry. In all cases, a BD FACSCanto (BD Biosciences, USA) cytometer was used.

### 2.5 Data Treatment

The software FlowJo (FlowJo LLC, USA) was used to process the flow cytometry raw data, whereas Origin (OriginLab Corporation, USA) was employed to obtain the graphs presented as well as to perform the associated statistical tests. In the box plots (boxes define the 25th and 75th percentiles), the central line is the median and the whiskers indicate one standard deviation.

## 3 Results

### 3.1 PEG–Heparin Hydrogels as Ink for 3D Printing

PEG–heparin hydrogels have recently been proposed as 3D scaffolds for the primary human cell cultures that the novel ACT requires, as they enhance immune cell proliferation of desired phenotypes.^17,18^ Nevertheless, this clinical application would require a scaling-up and automatization of the hydrogel fabrication procedure, as these cellular therapies involve large volumes of cell culture.

With this objective in mind, we assessed the feasibility of using preformed PEG–heparin hydrogels as ink. Consequently, we optimized the process of hydrogel formation to produce well-defined 3D scaffolds that were previously predesigned, using a 3D Discovery instrument (RegenHU Biosystem Architects, Switzerland). After an optimization process, the extrusion pressure set for the 3D printer was 1.2 bar, using a conic tip with an inner diameter of 27 G (0.36 mm).

**Figure 2:**
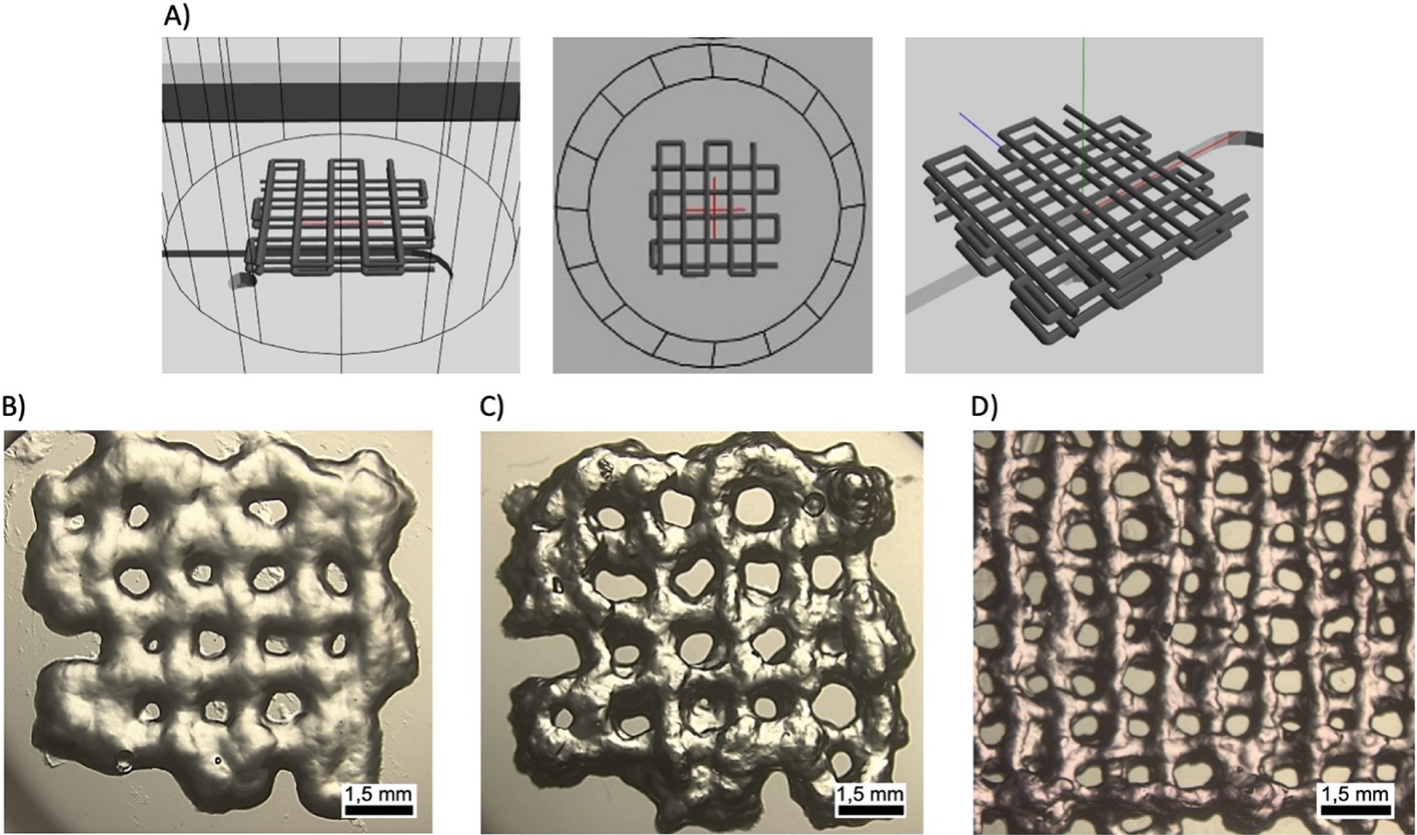
A) Schematic projections of the scaffold that were designed to optimize the 3D printing of PEG–heparin hydrogels. Microscope images of the resulting scaffolds printed with a preformed PEG–heparin hydrogel in PBS for B) 3.5 h and C) 1 day after mixing the components. D) Microscope image of a scaffold printed with a preformed PEG–heparin hydrogel in DMEM.

The 3D scaffold design was selected to optimize nutrient, gas, and waste exchange, aiming to maximize cell viability and proliferation. This design maintains the intrinsic hydrogel micrometer-scale porosity, whose efficacy was previously demonstrated.^17,18^ Moreover, a simple design was chosen, which is commonly used to assess novel materials, and consists of a four-layered grid with lines spaced 1.5 mm apart (Figure 2A). Its simplicity can also facilitate its translational potential and technology transfer options. With this purpose, PEG–heparin hydrogels were preformed and tested as ink for 3D printing at room temperature and different hydrogel formation times. 3.5 h after mixing the two components, 4-arm PEG thiol and maleimide-functionalized heparin, the sample had achieved enough viscosity to be printed (Figure 2B). However, the resulting scaffolds showed low consistency with poorly differentiated lines in the printed grid. To enhance their quality, the preformed hydrogels were subsequently used after 12 h of mixing, which resulted in suitable scaffolds for our purpose (Figure 2C). Finally, we also evaluated the possibility of printing the PEG–heparin hydrogel dissolved in cell media for potential cell-laden experiments. In this case, hydrogel formation immediately occurred, possibly due to the presence of less divalent ions, which facilitate the formation of disulfide bonds, thus reducing the thiols available for hydrogel formation.^31^ Interestingly, the resulting material could be readily printed (Figure 2D).

### 3.2 PEG–Heparin Printed Scaffolds for CD4+ T Cell Expansion

Once the printability of PEG–heparin hydrogels was demonstrated, 3D layered structures were printed and assessed as 3D scaffolds for primary human CD4+ T cell culture.

**Figure 3:**
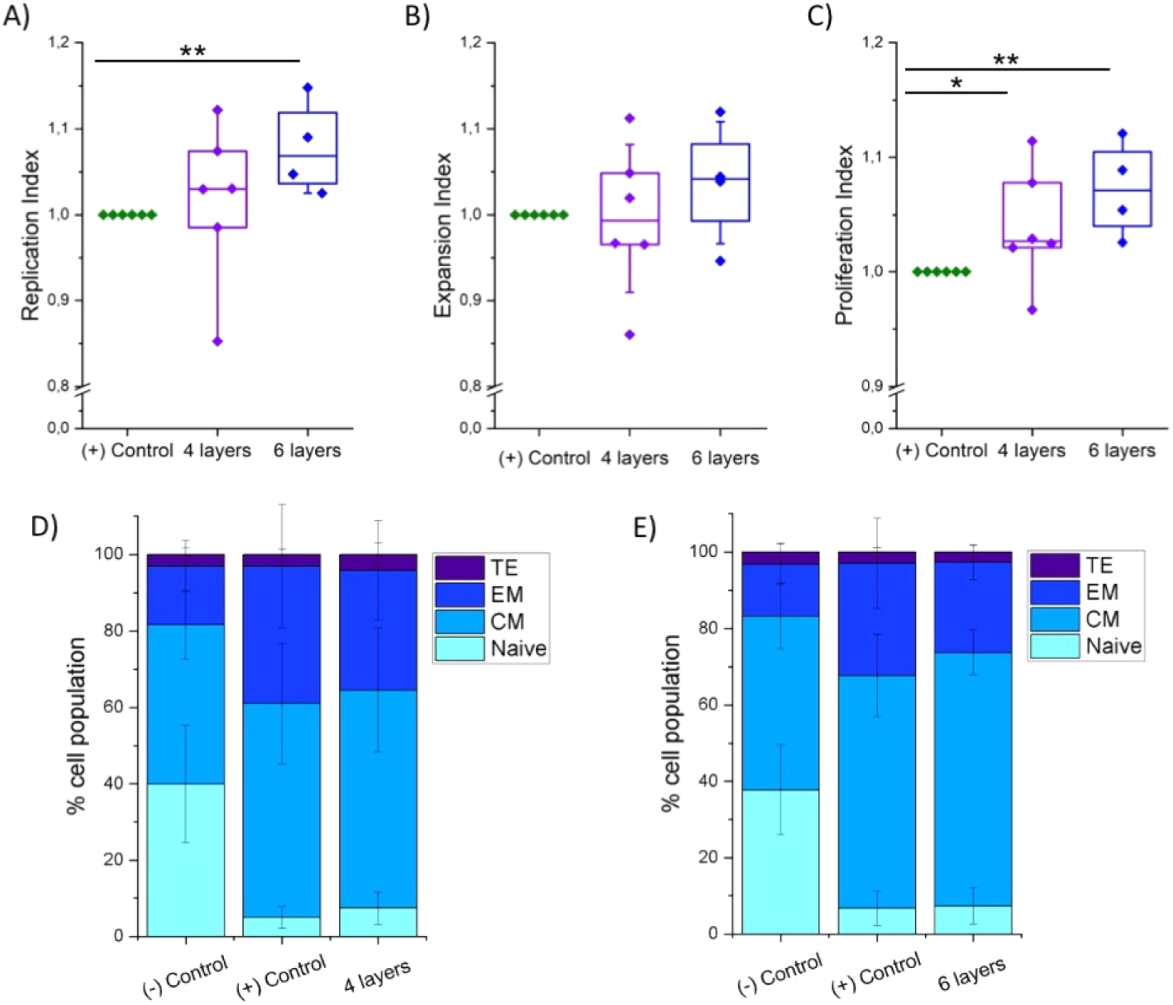
Normalized proliferation results of primary human CD4+ T cells cultured for 6 days in printed PEG–heparin hydrogels with 4 or 6 layers of height and in suspension (positive control): A) replication, B) expansion, and C) proliferation indexes (*N*_donors_ = 6). Statistical significance was determined by the Mann–Whitney U test (*p < 0.05 and **p < 0.01). Differentiation analysis of CD4+ T cells (effector, *T*_EFF_: TE; effector memory, *T*_EM_: EM; central memory, *T*_CM_: CM; naive, *T*_N_: naive) cultured in printed PEG–heparin hydrogels of D) 4 and E) 6 layers of height with their corresponding controls (*N*_donors_ = 6).

As a starting point, scaffolds consisting of 4 or 6 layers were produced with a separation of 1.5 mm between lines, employing ca. 35 and 50 μg of material, respectively. Then, primary human CD4+ T cells were seeded on them at a concentration of 10^6^ cells/ml for 6 days. Then, the proliferation, replication, and expansion indexes^32^ of samples and controls (cells seeded in suspension) were obtained by flow cytometry and compared (Figure 3A–C).

The four-layered printed PEG–heparin scaffold showed a slight tendency to enhance the proliferation indexes compared to the positive control, obtaining the only significant difference in the proliferation index with a normalized value of 1.03. On the other hand, the six-layered printed scaffolds exhibited higher statistically significant proliferation results, enhancing the proliferation and replication indexes by 7% (normalized value of 1.07) and by 4% for the expansion index. As expected, cell proliferation is positively influenced by the amount of hydrogel used; i.e., the higher the number of layers, the higher the proliferation parameters.

In the next step, the phenotype of the resulting T cells was analyzed for both types of 3D printed hydrogels 5 days after seeding. In particular, the cells were classified as naive (*T*_N_; CD45RO–/CD62L+), central memory (*T*_CM_; CD45RO+/CD62L+), effector (*T*_EFF_; CD45RO–/CD62L−), and effector memory (*T*_EM_; CD45RO+/CD62L−).^21^ For the four-layered hydrogels (Figures 3D and S1), a statistically significant increase in the percentage of the *T*_CM_ phenotype was obtained, together with a reduction of the *T*_EM_. Specifically, the median value of *T*_CM_ was 66% for the four-layered printed hydrogels and 61% for the positive control, compared to 45% for the negative control. Additionally, the *T*_EM_ mean values were 23% for the printed scaffold, compared to 30 and 14% for the positive and negative controls, respectively. Finally, the *T*_N_ cells suffered a significant decrease from 39% of the inactivated cells to 5 and 6% of cells cultured in suspension or using hydrogels, respectively, although no significant differences were obtained between activated cells. Although a similar trend was observed for the six-layered scaffolds, the observed differences were less prominent, especially for the *T*_CM_ phenotype (Figures 3E and S2).

### 3.3 Scaling-Up the Size of Hydrogels by 3D Printing

The use of PEG–heparin hydrogels has recently been demonstrated to improve immune cell culture in terms of both proliferation and differentiation,^17,18^ which are interesting results toward cellular therapies such as ACT. Nevertheless, these treatments require cultures in volumes that might be in the order of liters, while the results so far have been achieved with volumes below 1 mL. Thus, it is necessary to scale-up the size of the hydrogels to enable their clinical application. However, the increase in the size of the scaffolds will surely make the transfer of cells, nutrients, and gases through them more difficult.

**Figure 4:**
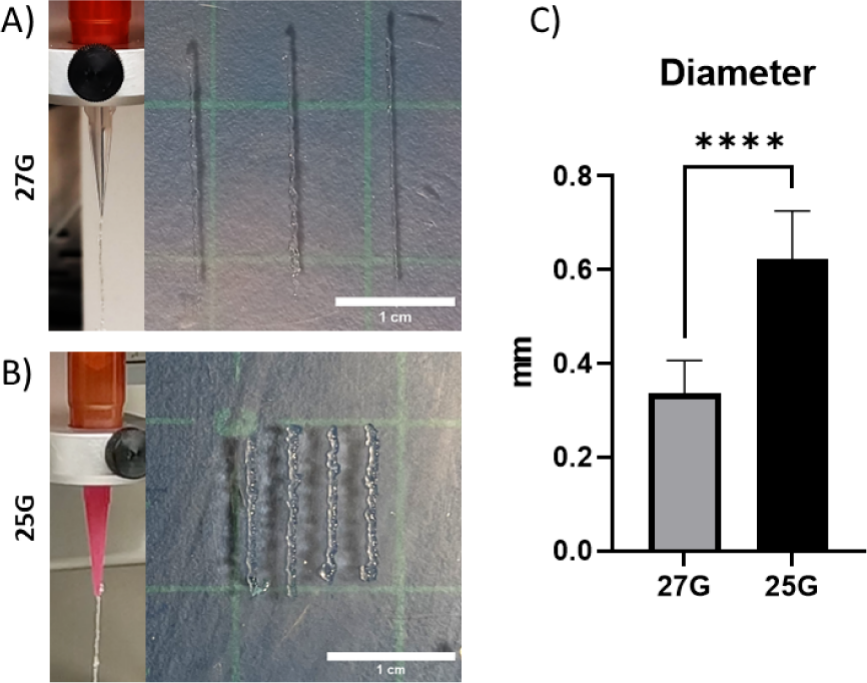
Representative images of the hydrogel extruded on air and 3D printed filaments with 27G (A) and 25G (B) needles (scale bar = 1 cm). C) Obtained diameters from 3D printed hydrogels for each needle.

**Figure 5:**
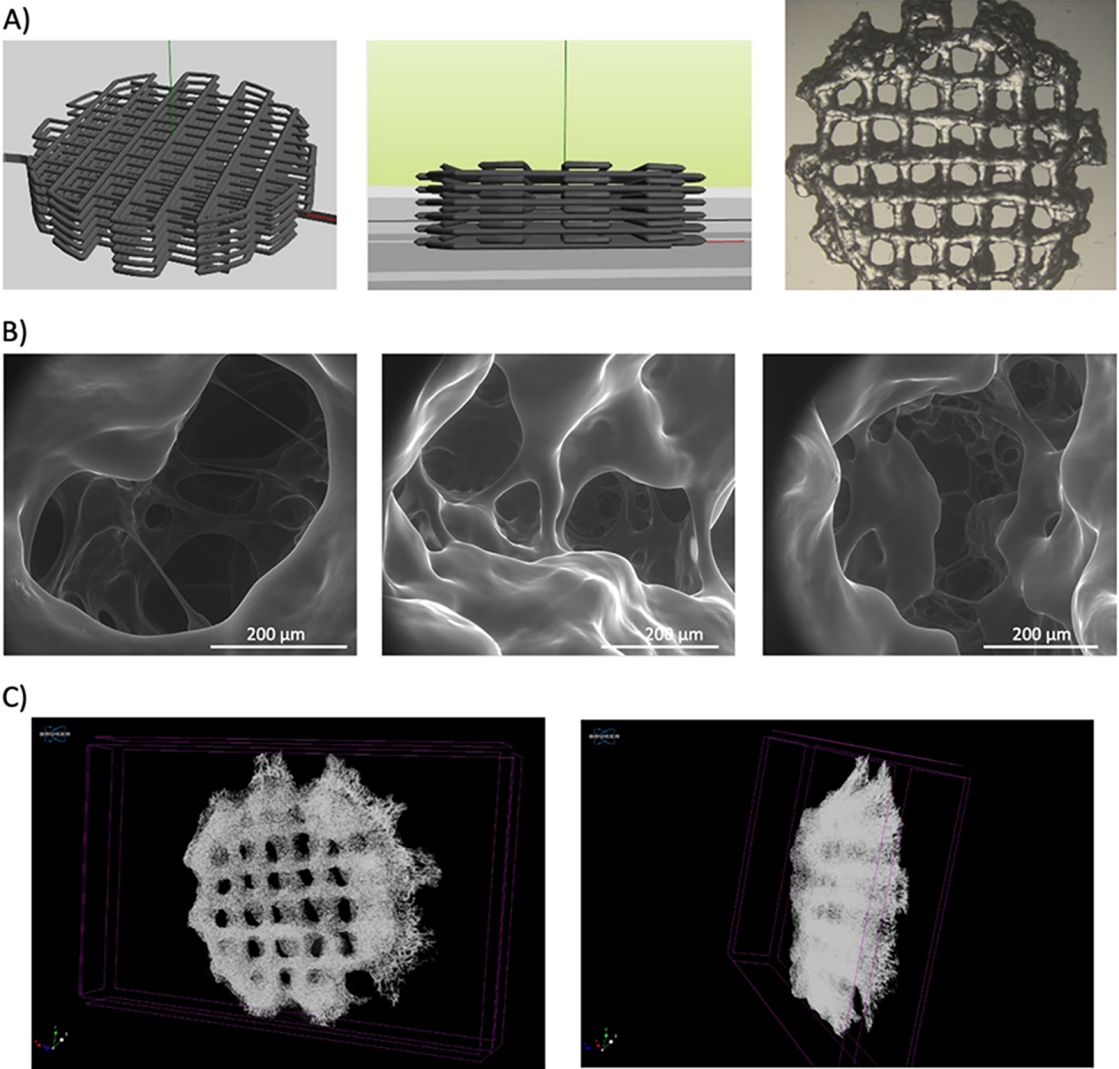
A) Schematic and photographic images of the 3D printed hydrogels designed to maximize the size of the scaffold, which has a diameter of 1 cm, and the amount of material used. B) SEM images of the cavities in the interior of the large 3D printed hydrogels. C) Top and lateral views of a large 3D printed hydrogel (diameter of 1 cm) obtained with X-ray microtomography imaging.

To overcome this issue, we studied the use of 3D printing to scale up the scaffolds’ size. Therefore, we selected a needle with a higher diameter (25G) and compared the printability to the previous one. In both cases, a continuous filament was obtained by extruding the preformed PEG–heparin hydrogel through the needles and printing segments (Figure 4A,B). As we expected, the diameter of filaments using a higher needle significantly increased (Figure 4C). In addition, the spreading ratio increased as well from 1.68 ± 0.35 (27G) to 2.49 ± 0.41 (25G), in both cases acceptable as they are between 1 and 3.^33^ Furthermore, the structure was modified by designing a circular grid with ten layers separated 1.5 mm apart between lines and arranged perpendicularly in an alternating manner (Figure 5A).

This new design allowed us to increase the amount of the printed material 10-fold. For further characterization of the new design, X-ray microtomography imaging and environmental scanning electron microscopy (SEM) were employed to measure the pore size and interconnectivity of the printed hydrogels (Figure 5B,C). As shown in the images, the layered structure can be seen as well as the internal structure of the hydrogel previously observed for the bulk hydrogels.^18^

Also, the mechanical properties of the scaffolds were determined by rheology (Figure S3) and compared with the nonprinted material.^18^ The storage modulus (*G*′) achieved for the printed hydrogel was 447 ± 34 Pa in comparison with 1.1 ± 0.1 kPa of the nonprinted hydrogel.^18^ As expected, the printed hydrogel loses part of its hardness when printed in comparison with the same material in a bulk hydrogel. However, the mechanical properties of both hydrogel types are comparable, and the printed ones offer better accessibility of the cultured cells to the inside of the material, providing a better opportunity to scale-up the use of this material compared to the bulk hydrogel.

**Figure 6:**
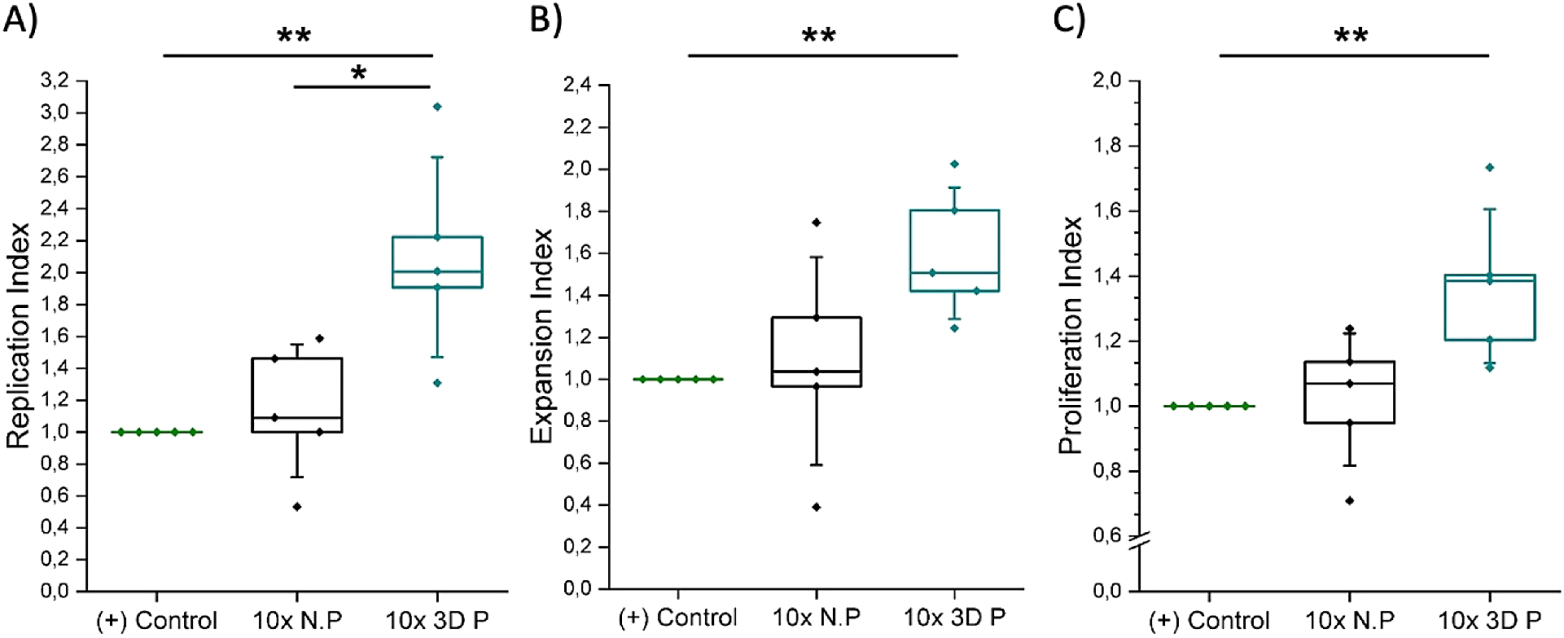
Normalized proliferation results of primary human CD4+ T cells cultured in suspension (positive control), large nonprinted PEG–heparin hydrogels (10x N.P), and large 3D printed PEG–heparin hydrogels (10x 3D P) 6 days after seeding: A) replication, B) expansion, and C) proliferation indexes (*N*_donors_ = 5). The Mann–Whitney U test was used to assess statistical significance (*p < 0.05 and **p < 0.01).

Finally, the large 3D printed hydrogels were used to culture primary human CD4+ T cells (Figure 6). To determine the benefits of the printed structure, we compared the results to a hydrogel of the same mass but not printed (bulk structure).

The replication index of the large 3D printed hydrogels showed a normalized mean value of 2, duplicating the amount of responding cells obtained with both the bulk hydrogels and the state-of-the-art suspension systems. The proliferation and expansion indexes also increased, with mean values of 1.4 and 1.5 for the 3D printed hydrogels in comparison with the values of 1.08 and 1.04 for the bulk hydrogels, respectively. It is also worth pointing out that 50 and 40% improvements were therefore observed compared to suspension systems. In summary, all the proliferation parameters were higher for cells seeded in the large 3D printed hydrogels than those seeded in suspension, and more interestingly, than the analogous bulk hydrogels, in contrast with the results obtained for smaller hydrogels (Figure S4). Consequently, this experiment confirmed our hypothesis about the importance of the 3D printing technique to scale-up the hydrogels to be translated to the clinics.

## 4 Conclusions

We demonstrated that PEG–heparin hydrogels can be successfully 3D printed, which opens up numerous potential applications, including cell-laden constructs. Moreover, the proliferation of primary human CD4+ T cells was enhanced in cells incubated in the printed scaffolds compared to suspension cultures, with higher rates for scaffolds of 6 layers in comparison with four-layered scaffolds. Additionally, these 3D printed hydrogels led to an increase in the percentage of *T*_CM_ cells on day 5, a phenotype associated with high efficacy in immunotherapies.

Finally, large 3D printed hydrogels were produced to assess the scalability of PEG–heparin laboratory hydrogels and, therefore, their potential use as 3D scaffolds for the immune cell culture needed for ACT. In these experiments, higher proliferation ratios were obtained for the 3D printed hydrogels in comparison with both the state-of-the-art suspension methods and the bulk hydrogels. These results can be explained by the enhanced transport of cells, waste, nutrients, and gases to the inner part of the 3D printed hydrogels compared with the bulk ones. Thus, we showed that combining the benefits of the material with the benefits of the 3D printing technique might result in PEG–heparin hydrogels that can be useful in the clinics.

## Data Availability

The data that support the findings of this study are available from the corresponding author upon reasonable request.

## Supporting Information Available

Differentiation analysis of primary human CD4+ T cells; mechanical properties of 3D printed hydrogels; 3D printing for small-sized hydrogels (PDF)
