# Supplementary material for: 3D Printing as a Strategy to Scale-Up Biohybrid Hydrogels for T Cell Manufacture
Source: ACS Appl Mater Interfaces. 2024 Sep 17;16(38):50139–46. doi: 10.1021/acsami.4c06183 (PMC11440455; doi:10.1021/acsami.4c06183)
Supplement: Supplementary file 1 — am4c06183_si_001.pdf [file am4c06183_si_001.pdf]

Supporting Information

3D Printing as a Strategy to Scale-Up Biohybrid Hydrogels for T Cell Manufacture

Eduardo Pérez del Río,[1,2] Sergi Rey-Vinolas,[3,4] Fabião Santos,[1,2] Miquel Castellote-Borrell,[1,5] Francesca Merlina,[1,5] Jaume Veciana,[1,2] Imma Ratera,[1,2] Miguel A. Mateos-Timoneda,[6] Elisabeth Engel,[2,3,4,*] Judith Guasch[1,2,5,*]

Corresponding authors: eengel@ibecbarcelona.eu (E.E.) and jguasch@icmab.es (J.G.)

[1]Department of Molecular Nanoscience and Organic Materials, Institut de Ciència de Materials de Barcelona (CSIC), Campus UAB, 08193 Bellaterra (Spain)

[2]Centro de Investigación Biomédica en Red de Bioingeniería, Biomateriales y Nanomedicina (CIBER-BBN), 28029 Madrid (Spain)

[3]IMEM-BRT Group, Department of Materials Science and Engineering, EEBE, Technical University of Catalonia (UPC), 08019 Barcelona (Spain)

[4]Institute for Bioengineering of Catalonia (IBEC), The Barcelona Institute of Science and Technology (BIST), 08028 Barcelona (Spain)

[5]Dynamic Biomaterials for Cancer Immunotherapy, Max Planck Partner Group, ICMAB-CSIC, Campus UAB, 08193 Bellaterra (Spain)

[6]Bioengineering Institute of Technology, Universitat Internacional de Catalunya (UIC), 08195 Sant Cugat del Vallès (Spain)

1. Differentiation analysis of primary human CD4+ T cells

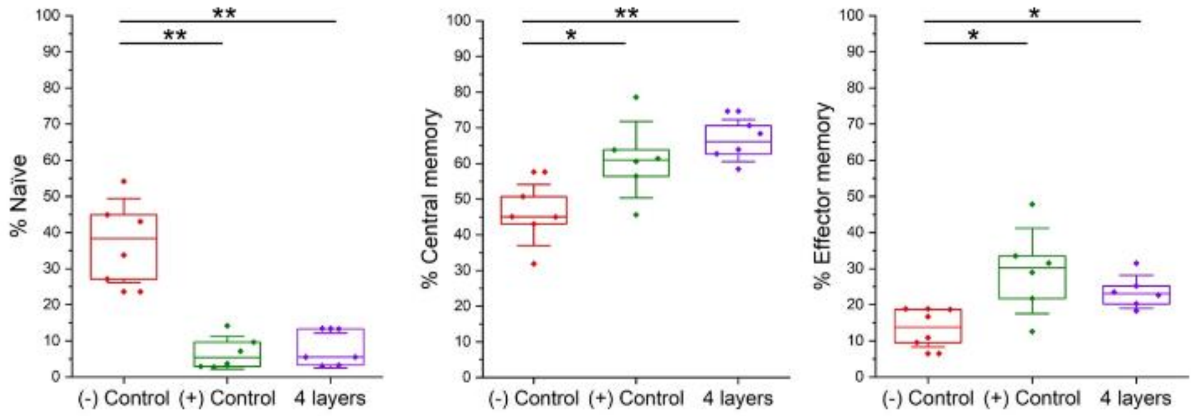

Figure S1. Differentiation analysis of primary human CD4+ T cells seeded in printed PEG-Hep hydrogels of 4 layers of height with their respective controls (N_{donors} = 6). Statistical significance was determined by the Mann-Whitney U test (*p<0.05, **p<0.01).

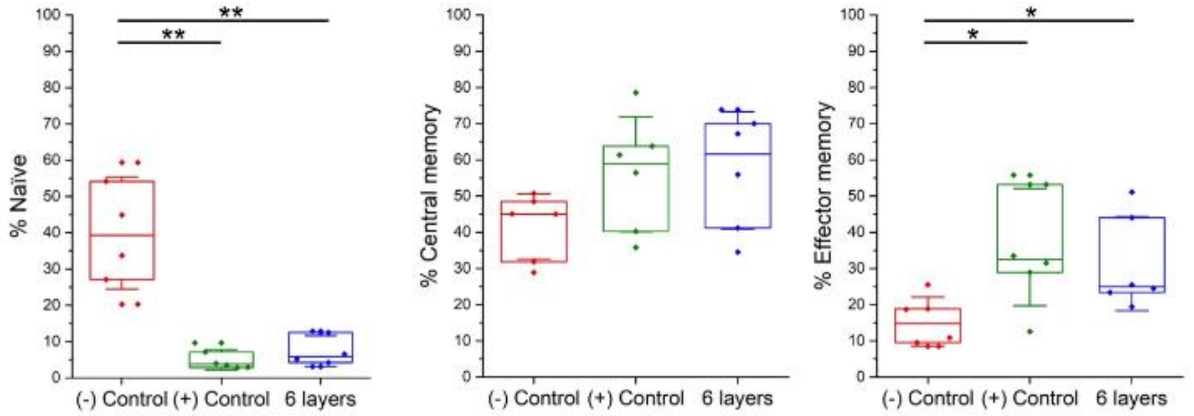

Figure S2. Differentiation analysis of primary human CD4+ T cells seeded in printed PEG-Hep hydrogels of 6 layers of height with their respective controls (N_{donors} = 6). Statistical significance was determined by the Mann-Whitney U test (*p<0.05, **p<0.01).

2. Mechanical properties of 3D printed hydrogels

The mechanical properties of the resulting scaffold were measured by rheology and compared with the non-printed material.[1] Strain sweeps were performed at a constant frequency of 1.0 Hz and the pressure was swept from 1 Pa to 50 Pa on the printed hydrogel and compared with the non-printed hydrogel (Figure S3). The linear behavior of the dynamic modulus (G′) of the printed hydrogel is within the strength range of 1–10 Pa, which is a lower value than the linear behavior from 10–100 Pa of the non-printed hydrogel.

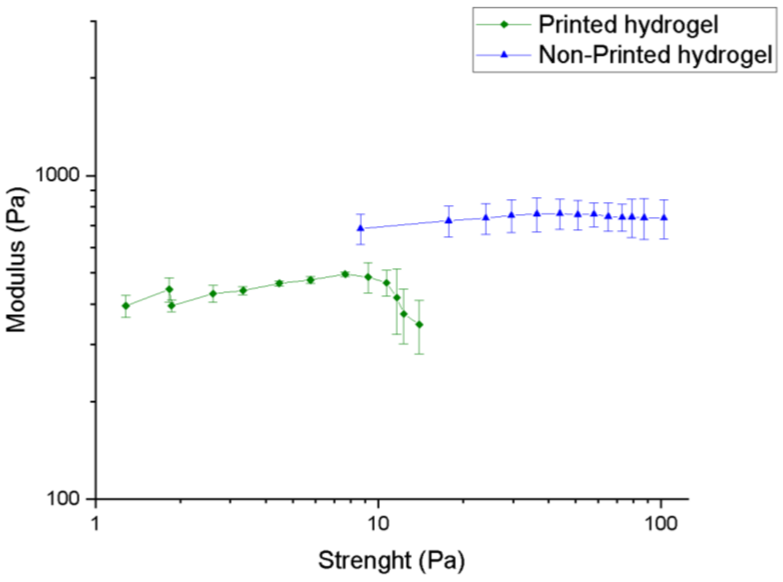

Figure S3. Strain sweeps at a constant frequency of 1.0 Hz to compare the mechanical properties of the hydrogel printed and non-printed.

The equilibrium shear modulus (G_e) achieved for the printed hydrogel was of 447 ± 34 Pa in comparison with the 1.1 ± 0.1 KPa of the non-printed hydrogel.

3. 3D printing for small size hydrogels

In these experiments, we compared the proliferation results obtained with 6-layer 3D printed hydrogels and with non-printed hydrogel (N.P. 3%wt) of the approximately the same mass (~50 µg). The results can be seen in Figure S4.

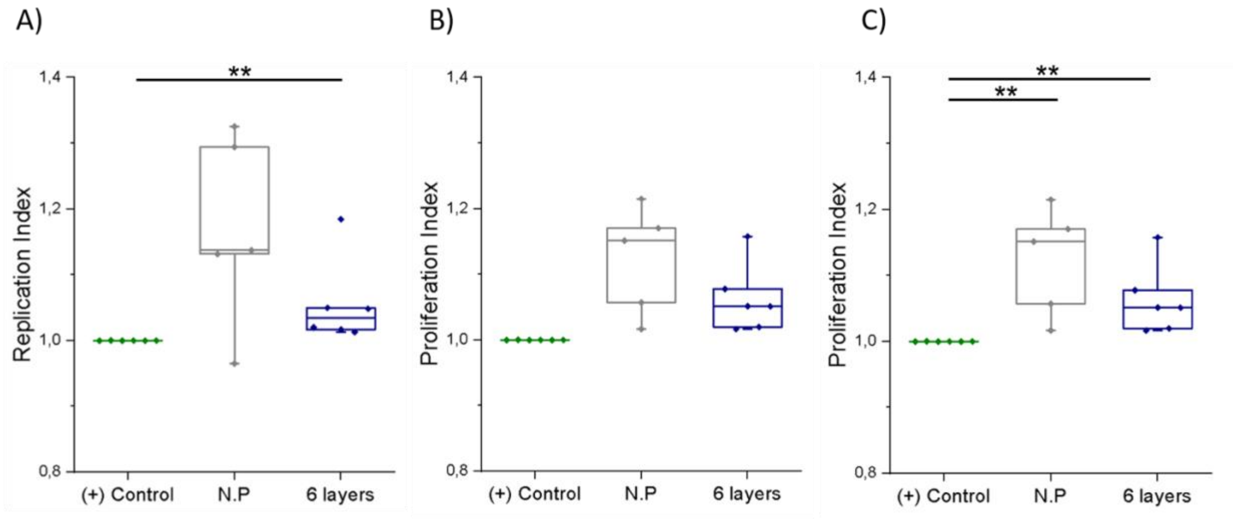

Figure S4. Normalized proliferation analysis of primary human CD4+ T cells seeded in suspension (positive control), non-printed (N.P., bulk) PEG-heparin hydrogels and 6-layer 3D printed hydrogels 6 days after seeding A) Replication, B) expansion, and C) proliferation indexes (N_{donors} = 6). Statistical significance was determined by the Mann-Whitney U test (*p<0.05, **p<0.01).

The non-printed PEG-heparin hydrogels improved the proliferation indexes ca. 15% (i.e. mean values of 1.15) compared to the positive control, although these changes were only statistically relevant in the case of the proliferation index. These results evidenced that, at this scale of production, the used non-printed hydrogels allow a better transfer of cells, waste, nutrients, and gases.

4. Reference

(1) Pérez del Río, E.; Santos, F.; Rodriguez, X. R.; Martínez-Miguel, M.; Roca-Pinilla, R.; Arís, A.; Garcia-Fruitós, E.; Veciana, J.; Spatz, J. P.; Ratera, I.; Guasch, J. CCL21-loaded 3D hydrogels for T cell expansion and differentiation. *Biomaterials* **2020**, *259*, 120313.
